# The Journal’s Work

**Published:** 1857-02

**Authors:** 


### THE JOURNAL'S WORK.

"I READ and re-read the contents of your invaluable Journal many times each month, and, taking it up from my writing table this morning, for certainly the twentieth reading of the December number, I learned its terms were one dollar in all cases, and not fifty cents to clergymen, as last year. If it were fifty cents, I considered it my duty to myself and the loved ones at home, who look to me for the necessaries of life, to send fifty cents, and so equally to send a dollar, or two, or three, if that be the price; for I am satisfied in my inmost soul, that I owe my life, and whatever I shall enjoy of health in years to come, to the instructions contained in your Journal. My experience is somewhat interesting in connection with your Journal, and my recovery from an advanced consumptive condition, after consulting and being under treatment with some of the most eminent physicians in New York and Boston. I grew continually worse, till, meeting with your Journal, I took the medicine therein prescribed—a *horse* and *garden*—and am now, I have every reason to believe, on the *sure road*, and nearly at the goal, of permanent health; and that with the continuation of my pulpit labors, though my society urged me to accept of my salary and rest for some months, which I did, while growing worse, by *Inhalation*, &c., &c. On adopting your medicine I commenced preaching again, and talking, on an average, four other days in the week on school committee business, in weekly meetings, and at funerals, and, in a short time, was *stronger* and heavier than for years before.

"Very sincerely, yours,

The above is dated December 31st, and while it is encouraging to us to be remotely instrumental in restoring a useful and efficient clergyman to his labors, it should inspire other clergymen with the ambition to get well *in the continuance of their labors.*

